# Five Trials, Five Different Questions: Design Heterogeneity Among Lipoprotein(a)-Lowering Cardiovascular Outcomes Trials After Lp(a)HORIZON

**DOI:** 10.7759/cureus.117424

**Published:** 2026-10-04

**Authors:** Humberto G Moreira, Jorge Nelson M Peres Filho, Luiz Eduardo P Marquesani, Mateus B De Almeida, Juliana G Lana

**Affiliations:** 1 Medical School, Federal University of Goiás, Goiânia, BRA; 2 Cardiovascular Medicine, Faculdade Atenas, Passos, BRA; 3 School of Medicine, Universidade de Uberaba, Uberaba, BRA; 4 School of Medicine, Master Education Institute President Antonio Carlos (IMEPAC), Araguari, BRA; 5 School of Medicine, Afya Ciencias Medicas, Ipatinga, BRA

**Keywords:** cardiovascular outcomes trial, lepodisiran, lipoprotein(a), muvalaplin, olpasiran, pelacarsen, registry-based study

## Abstract

Lipoprotein(a) (Lp(a)) is a genetically determined risk factor for atherosclerotic cardiovascular disease (ASCVD), widely regarded as causal on the basis of genetic and epidemiological evidence, for which no approved Lp(a)-lowering therapy exists. In September 2026, Novartis announced that Lp(a)HORIZON, evaluating the antisense oligonucleotide (ASO) pelacarsen and the most clinically advanced of five phase 3 cardiovascular outcomes trials (CVOTs) targeting Lp(a), had not met its primary composite endpoint. Key details, including the magnitude of Lp(a) lowering achieved, the result in the pre-specified higher-Lp(a) subpopulation, the effect estimate, and the safety profile, remain unpublished pending congress presentation. This review compares the design of all five phase 3 Lp(a)-lowering CVOTs, identified through a curated bibliography, a PubMed search for design and rationale publications, and searches of the ClinicalTrials.gov registry, and situates them within the wider landscape of Lp(a)-directed therapy. The four trials still running are not replications of the first. They differ from Lp(a)HORIZON and from one another in mechanism (ASO, small interfering RNA (siRNA), or oral small molecule), dosing interval, Lp(a) eligibility threshold, sample size, stage of disease at which they intervene, and, critically, in the composition of the primary endpoint: the two olpasiran trials use a coronary-only composite that excludes stroke, whereas Lp(a)HORIZON and, by its own nomenclature, MOVE-Lp(a) include it. A coronary-only composite and a stroke-inclusive composite do not estimate the same quantity, so effect estimates from these trials cannot be directly compared without accounting for endpoint composition, population, and follow-up. Five non-exclusive explanations for the neutral result are set out, together with the evidence bearing on each and what would distinguish them. A single neutral trial does not resolve the Lp(a) question, and the readouts expected between 2027 and 2031 will not resolve it jointly unless these design differences are held in view when the results are compared.

## Introduction and background

Elevated lipoprotein(a) (Lp(a)) is an independent, genetically determined risk factor for atherosclerotic cardiovascular disease (ASCVD) and calcific aortic valve disease [1]. Unlike low-density lipoprotein cholesterol (LDL-C), Lp(a) is largely refractory to lifestyle intervention and to established lipid-lowering drugs, and no therapy specifically targeting Lp(a) has yet been approved [1]. Mendelian randomization and genetic association studies have consistently supported a causal relationship between Lp(a) and cardiovascular risk [2,3], but this causal inference has, until now, never been tested directly by a completed cardiovascular outcomes trial (CVOT) of a targeted Lp(a)-lowering therapy.

Several classes of Lp(a)-lowering agents have since entered clinical development: antisense oligonucleotides (ASOs), small interfering RNAs (siRNAs), and, more recently, oral small-molecule inhibitors. Early-phase studies showed Lp(a) reductions exceeding 80-90% [4-9], and these agents have now progressed to large-scale phase 3 CVOTs designed to test whether lowering the biomarker reduces cardiovascular events. Lp(a)HORIZON, which evaluated pelacarsen, was the most clinically advanced of these programs and the first to report [10,11].

On September 4, 2026, Novartis announced that Lp(a)HORIZON did not meet its primary endpoint [10]. This is, to our knowledge, the first CVOT result available for any targeted Lp(a)-lowering therapy, and it arrives while four additional phase 3 CVOTs targeting the same biomarker, through three different mechanistic classes, remain ongoing [12]. The full dataset is not yet available, and this review does not attempt to explain a result no one has seen. Its purpose is the opposite and is served better now than later: to put on record, while the four remaining trials are still running, how they differ from Lp(a)HORIZON and from one another in mechanism, dosing interval, eligibility threshold, endpoint composition, and stage of disease, so that those differences are available as a reference frame when the results arrive rather than reconstructed afterwards to fit them.

The Lp(a)HORIZON result

Lp(a)HORIZON (NCT04023552) was a phase 3, randomized, double-blind, placebo-controlled trial of pelacarsen (80 mg subcutaneously, monthly) in 8,323 patients with established cardiovascular disease (prior myocardial infarction, ischemic stroke, or symptomatic peripheral artery disease) and elevated Lp(a) (≥70 mg/dL, approximately 149 nmol/L, with a pre-specified subpopulation analysis at ≥90 mg/dL, approximately 192 nmol/L) [11]. The primary endpoint was a composite of cardiovascular death, non-fatal myocardial infarction, non-fatal stroke, or urgent coronary revascularization requiring hospitalization, in an event-driven design targeting 993 adjudicated events over a minimum follow-up of 2.5 years and an anticipated total duration of approximately six years [11].

According to the official press release, Novartis's President of Development and Chief Medical Officer, Shreeram Aradhye, stated: "Although lower Lp(a) levels were observed with pelacarsen, the findings did not demonstrate that this translated into reduced cardiovascular risk in the overall study population. These are not the results we hoped for, but they provide important evidence that advances scientific understanding of the relationship between Lp(a) lowering and cardiovascular outcomes" [10].

Critically, as of this writing, the magnitude of Lp(a) reduction actually achieved in Lp(a)HORIZON, the result of the pre-specified higher Lp(a) subgroup (≥90 mg/dL), and the trial's safety profile have not been publicly disclosed; Novartis has stated only that full data will be presented at an upcoming medical congress, without a confirmed date [10]. Any discussion of why the trial failed is therefore necessarily provisional, and the discussion that follows should be read as hypothesis generation rather than explanation. Early commentary raised two complementary readings: that background cardiovascular risk in the enrolled population was already intensively treated, leaving little residual risk for an incremental Lp(a)-lowering effect to act upon, and that Lp(a) may behave partly as a marker of accumulated arterial injury rather than as a dominant driver of the next event, which would reopen a question that genetic and Mendelian randomization evidence had appeared to settle [13]. Both are examined below, alongside three further possibilities and one of our own.

## Review

Methodology

This narrative review addresses the phase 3 CVOTs of therapies developed specifically to lower Lp(a). Searches and data extraction were carried out between September 3 and September 11, 2026. Two sources were searched. PubMed was queried without date restriction for design and rationale publications and for the phase 1/2 reports of each agent, using the expression ("lipoprotein(a)" OR "Lp(a)") AND (pelacarsen OR olpasiran OR lepodisiran OR muvalaplin OR zerlasiran) AND (trial OR design OR rationale). ClinicalTrials.gov was searched in two complementary ways, so that the resulting set would not depend on knowing the agent names in advance: an agent-anchored search, entering pelacarsen OR olpasiran OR lepodisiran OR muvalaplin OR zerlasiran in the intervention field, and a target-anchored search, entering "lipoprotein(a)" OR "Lp(a)" in the condition and other-terms fields. Both were restricted to interventional studies of phase 3. The two result sets were combined and deduplicated. Each extracted attribute carries its provenance, distinguishing a peer-reviewed publication from the primary registry record and from a registry-derived secondary source; attributes absent from all three are recorded as not reported. These searches were complemented by a curated bibliography of the Lp(a)-lowering literature assembled by the authors and cross-checked against a recently published ClinicalTrials.gov-based landscape review, which applied an independent structured registry search on November 5, 2025, and likewise identified five CVOTs [12].

A record was included if it met five conditions: (i) randomized interventional design; (ii) phase 3 of clinical development; (iii) a primary endpoint defined as a composite of major adverse cardiovascular events (MACE) rather than a biochemical endpoint such as percentage change in Lp(a); (iv) a design powered for that clinical endpoint, meaning either an explicit event-driven stopping rule or a planned enrollment and follow-up duration of a magnitude conventionally used to test a MACE endpoint; and (v) an intervention designed specifically to achieve pharmacological Lp(a) reduction. Records were excluded if they were of phase 1 or 2, if the primary endpoint was biochemical, if the intervention lowered Lp(a) only as a secondary pharmacological effect, or if the record was an observational study, an expanded access record, or a duplicate registration. Six trials across five investigational agents were screened, and five were retained (Figure 1). The single exclusion was zerlasiran, for which no phase 3 CVOT had been registered at the time of searching.

**Figure 1:**
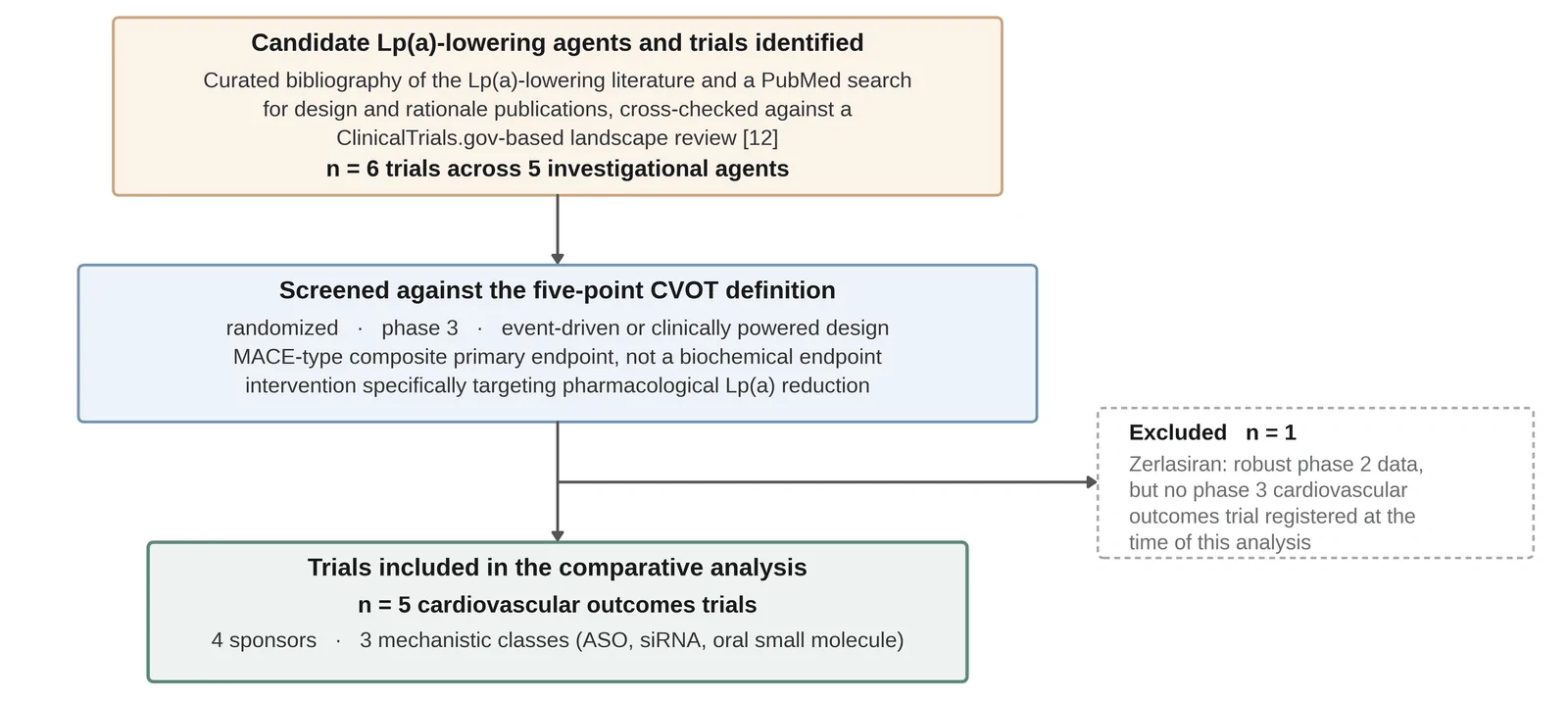
Selection process for the five included cardiovascular outcomes trials ASO, antisense oligonucleotide; CVOT, cardiovascular outcomes trial; MACE, major adverse cardiovascular event(s); siRNA, small interfering RNA; Lp(a), lipoprotein(a)

Four types of documents were used: the public ClinicalTrials.gov record of each included trial; the peer-reviewed design and rationale article, which exists for Lp(a)HORIZON only; the peer-reviewed phase 1/2 reports of each agent; and the sponsor's topline press release for Lp(a)HORIZON. Consensus guidance and one landscape review were used for context. The design attributes extracted for each trial were sponsor, phase, planned enrollment, Lp(a) eligibility threshold, prevention setting, primary endpoint definition, and study timeline. Extraction was performed independently by two authors, and the results were compared field by field, with disagreements resolved by consensus against the primary source. Attributes that could not be confirmed against a cited source are reported as not available rather than inferred. No patient-level data were used, and no ethics approval was required.

This search was structured but not systematic. It was not registered, did not follow Preferred Reporting Items for Systematic Reviews and Meta-Analyses (PRISMA), and used a single pre-specified expression per source rather than an exhaustive multi-database protocol. The review should be read accordingly. The five trials are compared descriptively. Differences in prevention setting, eligibility threshold, mechanism, and endpoint composition place them beyond the reach of quantitative synthesis, and no pooled estimate is attempted.

The five trials compared

Five phase 3 CVOTs met all five inclusion criteria (Table 1) [10,11,14-18]. All five test the same underlying hypothesis that pharmacologically lowering Lp(a) reduces cardiovascular events, but they hold almost no other design feature constant. They come from four sponsors and three mechanistic classes; they were launched across a three-year span, from late 2022 to late 2025; and their expected readouts are staggered even further, from a result already in hand to a completion date as distant as 2031.

**Table 1:** Comparative design characteristics of the five Lp(a)-lowering cardiovascular outcomes trials meeting the pre-specified CVOT inclusion criteria Thresholds are given in the units each trial uses, with an approximate equivalent in parentheses derived from the correspondence Lp(a)HORIZON reports for its own thresholds [11]; no single conversion applies across assays, since the relationship varies with apolipoprotein(a) isoform size [4]. On this basis, Lp(a)HORIZON enrolled at a numerically lower threshold than the four ongoing trials. Design attributes were extracted between September 3 and 11, 2026, from the ClinicalTrials.gov record of each trial [14-18], the Lp(a)HORIZON design publication [11], and the sponsor's topline press release [10]. Attributes not reported in those sources are marked as such rather than inferred. ASO, antisense oligonucleotide; CAD, coronary artery disease; CV, cardiovascular; CVOT, cardiovascular outcomes trial; Lp(a), lipoprotein(a); MACE, major adverse cardiovascular event(s); MI, myocardial infarction; PAD, peripheral artery disease; SC, subcutaneous; siRNA, small interfering RNA; TIA, transient ischemic attack

CVOT / Agent	Mechanism / Class	Sponsor	Phase / N	Lp(a) Threshold	Population (Prevention Setting)	Primary Endpoint	Start / Expected Completion	Status / Result
Lp(a)HORIZON pelacarsen NCT04023552 [10,11]	ASO SC monthly, 80 mg	Novartis	3 N = 8,323	≥70 mg/dL (subgroup ≥90 mg/dL) (~149 / ~192 nmol/L)	Secondary. Prior MI, ischemic stroke, or symptomatic PAD	Composite: CV death, non-fatal MI, non-fatal stroke, urgent coronary revascularization requiring hospitalization	Start date not reported in the sources consulted. Completion: minimum follow-up 2.5 y; event-driven (993 events, ~6 y)	Topline result announced 2026-09-04: primary endpoint neutral in the overall population. Full data (magnitude of Lp(a) reduction, ≥90 mg/dL subgroup, safety) not yet published; congress presentation pending
OCEAN(a)-Outcomes olpasiran NCT05581303 [14]	siRNA SC; dosing interval not reported in the sources consulted	Amgen	3 N = 7,297	≥200 nmol/L (~94 mg/dL)	Secondary. Prior MI or coronary revascularization + additional risk factor	Composite: coronary heart disease death, MI, urgent coronary revascularization	Start: 2022-12-14. Estimated completion: 2028-03-31	Ongoing (recruitment closed)
ACCLAIM-Lp(a) lepodisiran NCT06292013 [15]	siRNA SC; dosing interval not reported in the sources consulted	Eli Lilly	3 N = 17,300 (largest sample)	≥175 nmol/L (~82 mg/dL)	Mixed (primary + secondary). Documented CAD/stenosis, with or without a prior event	Reduction in MACE	Start: 2024-03-05. Estimated completion: 2029-03-31	Ongoing (recruitment closed)
MOVE-Lp(a) muvalaplin NCT07157774 [16,17]	Oral small molecule (only oral agent in this landscape)	Eli Lilly	3 N = 10,450	≥175 nmol/L (~82 mg/dL)	Secondary. Atherosclerotic event within the prior 10 years	Time to first MACE-4 event	Start: 2025-09-02. Planned treatment duration: 5.25 y [17]; completion date not reported in the registry record	Ongoing
OCEAN(a)-PreEvent olpasiran NCT07136012 [18]	siRNA SC; dosing interval not reported in the sources consulted	Amgen	3 N ≈ 11,000	≥200 nmol/L (~94 mg/dL)	Primary. Excludes prior atherothrombotic event (MI, stroke, TIA, acute limb ischemia)	Composite: coronary heart disease death, MI, urgent coronary revascularization	Start: 2025-08-22. Estimated completion: 2031-10-20	Ongoing (recruiting)

Lp(a)HORIZON, the trial that has now reported, is also the one whose design reflects the earliest stage of this field. It is the only ASO program in this landscape, the only one to define its Lp(a) threshold in mass units (mg/dL) rather than the molar units every other trial now uses, and it enrolls broadly across prior myocardial infarction, ischemic stroke, or symptomatic peripheral artery disease. The four trials still to come differ from it in several consistent respects. Three of them, OCEAN(a)-Outcomes (Olpasiran Trials of Cardiovascular Events and Lipoprotein(a) Reduction - Outcomes), ACCLAIM-Lp(a), and OCEAN(a)-PreEvent (Olpasiran Trials of Cardiovascular Events and Lipoprotein(a) Reduction to Prevent First Major Cardiovascular Events), enroll only patients with molar Lp(a) levels of 175-200 nmol/L, numerically stricter thresholds than Lp(a)HORIZON's once unit differences are considered [19]; three of the four use siRNA rather than antisense chemistry [6,7], and one, MOVE-Lp(a), abandons the injectable format altogether for an oral small molecule that blocks particle assembly rather than apolipoprotein(a) synthesis (Figure 2) [9]. Whether these mechanistic differences translate into differences in achieved Lp(a) lowering is a separate question, taken up below, and one that the available phase 1/2 data cannot settle by direct comparison. OCEAN(a)-PreEvent differs from every other trial in this landscape, Lp(a)HORIZON included, in testing the hypothesis in primary prevention: a population that, by design, excludes anyone who has already had a myocardial infarction, stroke, or acute limb event.

**Figure 2:**
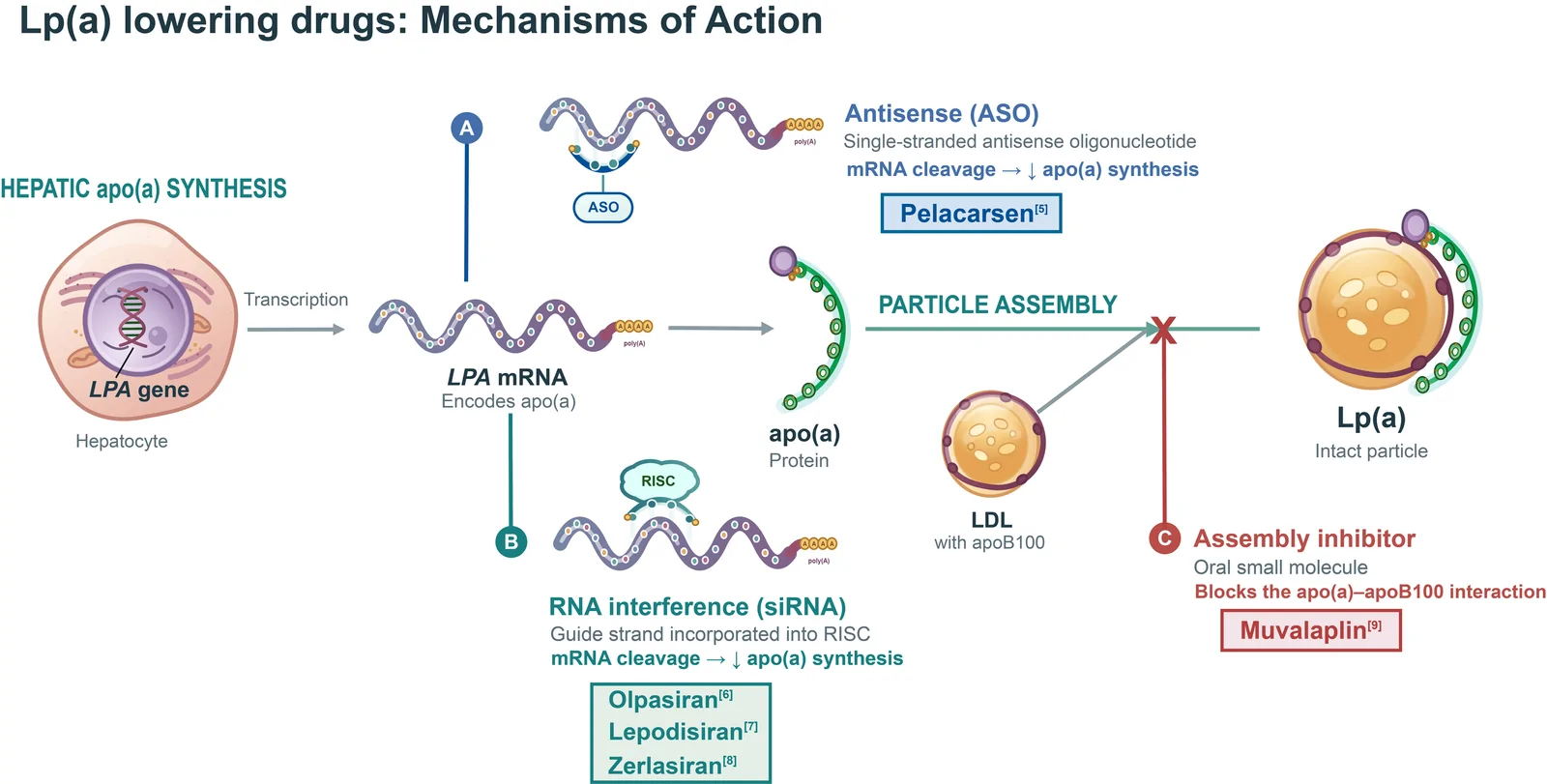
Pharmacological strategies for lowering Lp(a) Figure created by the authors using Adobe Illustrator version 30.3 (Adobe Inc., San Jose, USA). Lp(a) is assembled in the hepatocyte from apo(a), encoded by the LPA gene, and an LDL particle carrying apoB100: transcription of LPA yields mRNA, which is translated into apo(a), which is then covalently linked to apoB100 to form the intact particle. The agents in development interrupt this pathway at two points, giving three mechanistic classes. Panels A and B act on apo(a) synthesis and are given subcutaneously: an ASO (A) is single-stranded and hybridizes to LPA mRNA to direct its cleavage, whereas an siRNA (B) is double-stranded and loads a guide strand into RISC, which then cleaves the transcript catalytically, one reason siRNAs allow longer dosing intervals. Panel C acts downstream, after apo(a) has been made: muvalaplin, the only oral agent, binds apo(a) and blocks its interaction with apoB100, preventing particle assembly rather than reducing apo(a) production. Pelacarsen is the ASO; olpasiran, lepodisiran, and zerlasiran are siRNAs. Superscript numerals identify the phase 1/2 trial of each agent: pelacarsen [5], olpasiran [6], lepodisiran [7], zerlasiran [8], muvalaplin [9]. Zerlasiran is shown for mechanistic completeness only; no phase 3 cardiovascular outcomes trial had been identified for it at the time of this review, and it is not among the trials in Table 1. The diagram is schematic and not drawn to scale. apo(a), apolipoprotein(a); apoB100, apolipoprotein B-100; ASO, antisense oligonucleotide; LDL, low-density lipoprotein; LPA, the gene encoding apolipoprotein(a); Lp(a), lipoprotein(a); mRNA, messenger RNA; poly(A), polyadenylate tail; RISC, RNA-induced silencing complex; siRNA, small interfering RNA

A subtler but no less consequential divergence sits inside the primary endpoint itself, and it is easily missed unless the five definitions are compared component by component, which is the purpose of Table 2. Lp(a)HORIZON defines a four-component composite that includes stroke. Both Amgen-sponsored olpasiran trials, OCEAN(a)-Outcomes and OCEAN(a)-PreEvent, instead share an identical three-component, coronary-focused composite, coronary heart disease death, myocardial infarction, and urgent coronary revascularization, that excludes stroke altogether. MOVE-Lp(a) is identified in its registry record only as "MACE-4", a label that implies four components without naming them, and the components of ACCLAIM-Lp(a)'s composite are likewise not specified in the sources consulted. Both gaps are recorded as such in Table 2 rather than inferred from the endpoint's name.

**Table 2:** Lp(a)-related primary-endpoint assessment strategies across the five included cardiovascular outcomes trials Endpoint definitions were taken from the following sources: Lp(a)HORIZON from its design and rationale article and the sponsor's topline press release [10,11]; OCEAN(a)-Outcomes [14], ACCLAIM-Lp(a) [15], MOVE-Lp(a) [16,17], and OCEAN(a)-PreEvent [18] from their ClinicalTrials.gov records. Components not reported in those sources are recorded as such rather than inferred from the endpoint's name. CV, cardiovascular; CVOT, cardiovascular outcomes trial; Lp(a), lipoprotein(a); MACE, major adverse cardiovascular event(s); MI, myocardial infarction; OCEAN(a)-Outcomes, Olpasiran Trials of Cardiovascular Events and Lipoprotein(a) Reduction - Outcomes; OCEAN(a)-PreEvents, Olpasiran Trials of Cardiovascular Events and Lipoprotein(a) Reduction to Prevent First Major Cardiovascular Events

CVOT / Agent	Primary Endpoint Components	Endpoint Design	Distinguishing Note
Lp(a)HORIZON, pelacarsen [10,11]	4 components: CV death + non-fatal MI + non-fatal stroke + urgent coronary revascularization requiring hospitalization	Event-driven: 993 events confirmed by a clinical endpoint adjudication committee; tested in the overall population and in the Lp(a) ≥90 mg/dL subgroup, with multiplicity control for both tests	Only trial with stroke in the primary composite and with a formal test in a higher-Lp(a) subgroup
OCEAN(a)-Outcomes, olpasiran [14]	3 components: coronary heart disease death + MI + urgent coronary revascularization (no stroke)	Event count and statistical power not reported in the sources consulted	Coronary-focused endpoint, without a stroke component
ACCLAIM-Lp(a), lepodisiran [15]	Described generically as "reduction of major adverse cardiovascular events (MACE)"; exact components not reported in the sources consulted	Not reported in the sources consulted	Total N includes an addendum of ~1,700 participants to explore an alternative dosing regimen (pharmacodynamic sub-study within the CVOT itself)
MOVE-Lp(a), muvalaplin [16,17]	Termed "MACE-4" (4 components, by nomenclature); exact components not reported in the sources consulted	Time to first event; planned treatment duration 5.25 y [17]	Only oral agent; "MACE-4" nomenclature suggests stroke inclusion, to be confirmed in the official registry
OCEAN(a)-PreEvent, olpasiran [18]	3 components: coronary heart disease death + MI + urgent coronary revascularization (no stroke); identical to OCEAN(a)-Outcomes	Not reported in the sources consulted	Same endpoint definition as OCEAN(a)-Outcomes (same sponsor/molecule), but in primary prevention

These are not incidental design details. Each of the remaining trials alters at least one variable relative to Lp(a)HORIZON, whether a more potent mechanism, a stricter eligibility threshold, a narrower endpoint, or an earlier point in the disease course, and each therefore tests a distinguishable version of the same hypothesis. Which of these variations, if any, proves decisive is what the next several years of readouts will establish.

A third source of non-comparability sits in the statistical architecture of these trials rather than in their populations or endpoints. Lp(a)HORIZON is event-driven, continuing until 993 adjudicated primary events have accrued, and it is the only trial in this group known to carry a formal multiplicity-controlled test in a second population, the Lp(a) ≥90 mg/dL subgroup [11]. The four ongoing trials do not report event targets or power assumptions in their registry records, so it cannot be determined whether they are event-driven or duration-anchored, nor what alpha is allocated to any subgroup or secondary hypothesis. This matters in three ways. An event-driven trial and a duration-anchored trial of the same agent estimate treatment effect over different amounts of accrued exposure, so a shorter fixed-duration trial may be underpowered even with a larger sample. A trial that spends alpha on a subgroup has less available for its primary comparison, so an apparently modest primary result may reflect the multiplicity plan rather than the treatment. And when event rates fall below projection, as is plausible in contemporary secondary-prevention populations treated to low LDL-C levels, an event-driven trial extends in time whereas a duration-anchored trial simply loses power. None of this can be resolved from the public records, and we flag it as information that should be sought before any of these trials is compared with another.

The wider Lp(a)-lowering landscape

These five trials sit at the end of a much longer arc, and the reason they exist at all is recent. Lp(a) has been measurable for decades and, until the last few years, effectively unmodifiable. Lifestyle intervention does not lower it; statins do not lower it and may raise it modestly; proprotein convertase subtilisin/kexin type 9 inhibitors and mipomersen lower it by roughly 20% to 30%, an effect too small to test the hypothesis that Lp(a) reduction changes outcomes [1,4]. Only with RNA-directed approaches, which reduce Lp(a) by more than 80%, did a reduction of a magnitude worth testing become achievable, which is why the first CVOT of a targeted agent was reported in 2026 rather than a decade earlier [4]. Current consensus guidance was written for that gap: in the absence of a specific therapy, it recommends measuring Lp(a) at least once in adult life for risk stratification, managing global cardiovascular risk more intensively when it is elevated, and reserving lipoprotein apheresis for the rare patient with very high Lp(a) and progressive disease despite optimal treatment [1].

Not every agent that lowers Lp(a) substantially has reached a CVOT, and that attrition is itself part of the landscape. Zerlasiran, a siRNA that produced a placebo-adjusted, time-averaged reduction of 85.6% over 36 weeks in phase 2 [8], has no phase 3 CVOT identified at the time of writing and is therefore excluded from the comparison here despite pharmacodynamic data comparable to agents that are being tested. The five agents that did advance span three mechanistic classes, ASO, siRNA, and an oral inhibitor of particle assembly, and their phase 1/2 pharmacodynamic profiles differ in depth, duration, and route [5-9]. A broader survey of investigational Lp(a)-lowering therapies, including earlier-phase programs outside the scope of this review, has been published separately [12]. What distinguishes the five trials examined here is not that they are the only Lp(a)-directed programs, but that they are the only ones currently positioned to answer whether lowering Lp(a) changes what happens to patients.

Why the first trial may have been neutral

A neutral CVOT for the first targeted Lp(a)-lowering therapy to reach a primary readout is a sobering result for a field that had accumulated more than a decade of genetic and epidemiological evidence supporting Lp(a) as a causal, modifiable target. It is tempting to read Lp(a)HORIZON as a verdict on that hypothesis. The comparison presented above argues against that reading for a specific reason: the four trials still running are not replications of Lp(a)HORIZON. They differ from it and from one another in the mechanism used, the interval between doses, the eligibility threshold, the composition of the primary endpoint, and the stage of disease at which they intervene. Whatever Lp(a)HORIZON turns out to have shown, it is not obvious that any of the four is testing the same proposition.

Five explanations for the neutral result can be constructed from what is currently public. None can be preferred over the others on present evidence, and we set them out not to adjudicate between them but to specify what each would predict and which of the ongoing trials would bear on it (Table 3). Four derive from the design landscape characterized here. The fifth is our own conjecture and is offered as such.

**Table 3:** Five candidate explanations for the neutral Lp(a)HORIZON result, the evidence currently bearing on each, and what would distinguish them Table created by the authors. The five explanations are not mutually exclusive and are not ranked; on the currently available evidence, none can be preferred over the others, and the table is intended to specify what each would predict rather than to argue for any one of them. Explanations 1 to 4 derive from the design landscape characterized in Tables 1-2 [10,11,14-18]; explanation 5 is the authors' own conjecture and is supported only by analogy. apo(a), apolipoprotein(a); Lp(a), lipoprotein(a); LDL-C, low-density lipoprotein cholesterol; siRNA, small interfering RNA; OCEAN(a)-Outcomes, Olpasiran Trials of Cardiovascular Events and Lipoprotein(a) Reduction - Outcomes; OCEAN(a)-PreEvents, Olpasiran Trials of Cardiovascular Events and Lipoprotein(a) Reduction to Prevent First Major Cardiovascular Events

Candidate Explanation	What Supports It Now	Which Ongoing Trial Bears on It	What Would Confirm or Refute It
Insufficient magnitude or persistence of Lp(a) lowering	Phase 1/2 data show larger reductions for the siRNA agents, at 12-24 week intervals, than for pelacarsen given monthly. Pelacarsen's achieved phase 3 reduction is undisclosed.	OCEAN(a)-Outcomes, ACCLAIM-Lp(a), OCEAN(a)-PreEvent (all siRNA)	Publication of Lp(a)HORIZON's achieved reduction and its variation across the dosing interval; benefit in a trial achieving deeper or more sustained lowering at comparable baseline Lp(a).
Intervention too late in the disease course	Reasoning only. Lp(a)HORIZON enrolled established atherosclerotic disease with a minimum follow-up of 2.5 y against an exposure thought to act across decades.	OCEAN(a)-PreEvent (the only primary-prevention trial)	Benefit in primary prevention where secondary-prevention trials show none, at comparable achieved Lp(a) reduction.
Background cardiovascular risk already tightly controlled	Contemporary secondary-prevention populations reach substantially lower LDL-C than the cohorts in which Lp(a)'s risk associations were characterized.	All four, to differing degrees, given their staggered initiation dates	Published baseline characteristics and event rates for Lp(a)HORIZON against those of the ongoing trials; effect modification by baseline LDL-C or by achieved event rate.
Whole-particle reduction may not target the causal component	The Lp(a) particle carries distinct lipid, pro-inflammatory, and antifibrinolytic moieties; every agent here lowers the whole particle proportionally.	MOVE-Lp(a) (muvalaplin blocks particle assembly rather than apo(a) synthesis)	Divergent clinical results between assembly inhibition and synthesis suppression at equivalent measured Lp(a) reduction.
Inflammatory pathway redundancy (our conjecture)	No direct support. Argued by analogy to the ceiling seen with interleukin-1β blockade and to the population-dependence of colchicine.	None directly; all five agents act on Lp(a) rather than on inflammation	Inflammatory biomarkers as effect modifiers in patient-level data from these trials; a trial pairing an Lp(a)-lowering agent with anti-inflammatory therapy.

Hypothesis 1: insufficient magnitude or persistence of Lp(a) lowering

The agents in this landscape do not lower Lp(a) to the same degree, on the same time course, or at the doses carried into phase 3. In its phase 2 dose-ranging study, pelacarsen at 20 mg subcutaneously weekly reduced Lp(a) by a mean of 80% from baseline over six months [5], but Lp(a)HORIZON used a different regimen, 80 mg monthly [11], whose achieved reduction has not been disclosed [10]. The siRNA agents reported larger placebo-adjusted reductions at longer intervals: olpasiran, at 75 to 225 mg every 12 weeks, exceeded 95% at week 36 [6]; lepodisiran, at a pooled 400 mg dose, achieved a time-averaged reduction of 93.9% from day 60 to day 180 [7]; and zerlasiran, at 450 mg every 24 weeks, achieved a time-averaged reduction of 85.6% over 36 weeks [8]. Muvalaplin, the only oral agent, given at 240 mg daily for 12 weeks, reduced Lp(a) by 85.8% on an intact-particle assay and by 68.9% on an apolipoprotein(a)-based assay [9]. Regimens, timepoints, and assays differ enough across these studies that no efficacy ranking can be drawn from them, and we do not offer one. The relevant question is whether what matters is the depth of lowering, its persistence between doses, or the cumulative exposure avoided over the treatment period, which is the quantity atherosclerosis is generally held to respond to. On that view, an agent dosed monthly and one dosed every 12 weeks are not distinguished by their nadirs but by the area under their Lp(a)-time curves, and pelacarsen's position on that measure is unknown until its phase 3 pharmacodynamic data are released.

Hypothesis 2: intervention too late in the disease course

Lp(a)HORIZON enrolled patients with established atherosclerotic disease and treated them for a minimum of 2.5 years within an approximately six-year event-driven design [11], against an exposure thought to act cumulatively across decades [2,3]. If Lp(a) contributes mainly to the initiation and early progression of plaque rather than to the behavior of established lesions, a secondary-prevention trial of this duration may be structurally underpowered to detect benefit, however much Lp(a) is lowered. OCEAN(a)-PreEvent is the only trial in this landscape designed to intervene before a first atherothrombotic event (Table 1), which makes it the natural test of this explanation.

Hypothesis 3: background cardiovascular risk already tightly controlled

Contemporary secondary-prevention populations are treated to substantially lower LDL-C than the cohorts in which Lp(a)'s risk associations were originally characterized, and early commentary on Lp(a)HORIZON suggested that its population was well controlled in this respect [13]. The residual risk available for an incremental Lp(a)-lowering effect to act upon may therefore be smaller than the epidemiology anticipated. A related possibility is that event accrual was slower than planned, which would reduce power independently of any true treatment effect. Neither can be assessed until the trial's baseline characteristics and event curves are published. Both are properties of trial conduct and of the treated population rather than of the biological target, and both may differ across trials initiated several years apart.

Hypothesis 4: whole-particle reduction may not target the causal component

That the Lp(a) particle is more atherogenic than LDL per particle is well supported: an apolipoprotein B-based genetic analysis estimated its atherogenicity at approximately 6.6-fold (95% CI, 5.1-8.8) that of low-density lipoprotein (LDL) on a per-particle basis [20]. That estimate is made for the intact particle and cannot apportion risk among its parts, which is the difficulty. The particle carries at least three biologically distinct contributions: a cholesterol-carrying, LDL-like core; a pro-inflammatory component, mainly oxidized phospholipids bound to apolipoprotein(a); and a less consistently supported antifibrinolytic component related to apolipoprotein(a)'s homology with plasminogen. Every agent here lowers the whole particle proportionally, and none isolates a component. Muvalaplin is a partial exception in mechanism: by blocking apolipoprotein(a)-apolipoprotein B assembly rather than suppressing hepatic apo(a) production, it could in principle leave more free apo(a) in circulation, a distinction of unestablished clinical relevance. If the components contribute unequally, a strategy built on whole-particle reduction could underperform one able to target the causal moiety, which none of these agents can currently do.

A fifth hypothesis: inflammatory pathway redundancy

We propose, as a conjecture of our own rather than as an established mechanism, that part of Lp(a)'s epidemiological association with cardiovascular risk may run through inflammatory pathways only partially and non-uniquely coupled to Lp(a) particle number, a redundancy that would limit the yield of any strategy lowering Lp(a) without addressing the inflammatory drivers it shares with atherosclerosis more broadly. Lp(a) carries the majority of circulating oxidized phospholipids, and its pro-inflammatory properties are attributed in part to them [1]. The link is not merely inferential: lipid species enriched on the Lp(a) particle drive monocyte secretion of interleukin-6, interleukin-8 and interleukin-1β, partly through the NLRP3 inflammasome, and increase monocyte transendothelial migration; in the same work, pelacarsen lowered the circulating concentration of those species in patients with elevated Lp(a) [21].

That last finding is double-edged. It establishes that Lp(a) lowering does reach these mediators, which means the redundancy we propose must lie in pathways it does not reach. The clinical precedent for intervening on the inflammatory axis directly is instructive and unflattering. In CANTOS (Canakinumab Anti-inflammatory Thrombosis Outcomes Study), canakinumab reduced cardiovascular events at 150 mg every three months (hazard ratio, 0.85; 95% CI, 0.74-0.98; P=0.021), the only one of three doses to meet the trial's pre-specified multiplicity-adjusted threshold; neither 300 mg (hazard ratio, 0.86; 95% CI, 0.75-0.99; P=0.031) nor 50 mg (hazard ratio, 0.93; 95% CI, 0.80-1.07; P=0.30) did so, and the drug increased fatal infection [22]. Colchicine, acting further downstream, has produced a signal that depends on the population: benefit after myocardial infarction in COLCOT (Colchicine Cardiovascular Outcomes Trial) [23] and in chronic coronary disease in LoDoCo2 (Low-Dose Colchicine 2) trial [24], but a null result in an acute myocardial infarction population in CLEAR-SYNERGY (Colchicine and Spironolactone in Patients with Myocardial Infarction/SYNERGY Stent Registry) trial (hazard ratio, 0.99; 95% CI, 0.85-1.16; P=0.93) [25].

If broad anti-inflammatory strategies encounter a ceiling of this kind, a single upstream intervention on Lp(a) particle number might plausibly encounter a similar one, consistent with several partially redundant pathways converging on the same endpoint. No trial in this landscape tests this directly, since all five agents act on Lp(a) rather than on inflammation. Testing it would require pairing an Lp(a)-lowering agent with anti-inflammatory therapy, or examining inflammatory biomarkers as effect modifiers within the existing trials once patient-level data become available.

Taken together, these hypotheses do not dismiss the possibility, raised in early commentary, that Lp(a) behaves partly as a marker rather than as a fully actionable causal driver in some clinical contexts [13]. They argue that a single trial cannot settle the matter, and that the four remaining CVOTs, reporting between approximately 2027 and 2031, will not settle it jointly either unless their design differences are held in view when their results arrive. Recording those differences now, before the results exist to be reconciled with them, is the purpose of this review.

Implications for practice

For the clinician seeing a patient with elevated Lp(a) next week, the practical consequences of this result are narrower than the headlines suggest. Lp(a)HORIZON tested a drug, not the measurement, and nothing in a neutral pharmacological trial invalidates the epidemiological and genetic evidence that elevated Lp(a) identifies patients at higher lifetime cardiovascular risk. Measuring Lp(a) at least once in adult life, as recommended by current consensus guidance, remains reasonable on risk-stratification grounds [1]. What changes is the counselling that follows it. Patients who have been told, in many cases for several years, that a targeted therapy was imminent should be given an accurate account: the first such therapy to complete a CVOT did not reduce events in the population studied, four further trials are ongoing, and no Lp(a)-lowering agent is approved or expected to be approved on the strength of currently available evidence. In the meantime, the management of a patient with high Lp(a) is unchanged and consists of intensive control of every modifiable risk factor that can be treated, with the elevated Lp(a) level serving as a reason to set more ambitious targets rather than as a treatable entity in itself; lipoprotein apheresis remains an option in the rare patient with very high Lp(a) and progressive disease despite optimal therapy [1]. Clinicians enrolling patients in the four ongoing CVOTs should also be able to explain why those trials remain worth completing, which is the substance of the comparison presented here.

Implications for future trial design

Three design practices would make the next generation of Lp(a) trials more interpretable than the current ones. First, trials should report cumulative Lp(a) exposure over the treatment period, ideally as area under the Lp(a)-time curve, and not percentage change from baseline alone. If atherosclerosis responds to cumulative exposure, percentage change at a single timepoint is a poor summary of what a patient actually received, and it is the quantity that would make a monthly and a quarterly regimen comparable. Second, primary composite endpoints should be harmonized, or at minimum their components reported separately with enough granularity that a coronary-only and a stroke-inclusive composite can be reconstructed from the same dataset; without this, the trials cannot be pooled and can barely be compared. Third, baseline Lp(a) should be pre-specified as an effect modifier rather than examined post hoc, since the hypothesis that benefit requires a large absolute reduction predicts a gradient of effect across the baseline range, and that gradient is testable only if the analysis is planned in advance. We would add that publication of achieved Lp(a) concentrations across the dosing interval, rather than at trough or at a single visit alone, should become routine for agents whose dosing intervals differ as widely as these do.

Limitations of this review

Four limitations bear on how this review should be read. First, although extraction was performed independently by two authors and reconciled by consensus, for the four ongoing trials a subset of design attributes was read from registry-derived secondary sources reproducing the registry record rather than from a direct query of the ClinicalTrials.gov application programming interface; these should be re-verified before the data are used for any more granular analysis. Second, Lp(a)HORIZON's quantitative results, including the effect estimate, the pre-specified Lp(a) ≥90 mg/dL subpopulation, the achieved Lp(a) reduction, and the safety data, remain unpublished, so the five explanations in Table 3 are stated as competing predictions and none can currently be preferred on evidential grounds. Third, the pharmacodynamic figures cited for each agent (Hypothesis 1) come from different phase 1/2 trials with different regimens, timepoints, and Lp(a) assays, and, for pelacarsen, from a lower and more frequent dose than the one used in Lp(a)HORIZON; they are an approximate, non-head-to-head picture of achievable potency and not a validated ranking. Fourth, the inflammatory-redundancy hypothesis is our own conjecture rather than an established mechanism, and the canakinumab and colchicine trials cited in its support describe different agents in different populations and do not themselves demonstrate the proposed redundancy [22-25]. This is a narrative review built on a structured comparison of registry records and design publications. It follows an explicit, stated scope (Figure 1) but does not meet the methodological standards of a systematic review and should not be read as one.

## Conclusions

Lp(a)HORIZON's neutral primary result is the first word on whether pharmacological Lp(a) lowering reduces cardiovascular events, and it will not be the last. The four trials that follow differ from it in mechanism, dosing interval, eligibility threshold, endpoint composition, and prevention setting, by margins large enough that none of them is a replication and that their results cannot be predicted from this one. They are better understood as five related questions than as one question asked five times. Until pelacarsen's full dataset is presented, the Lp(a)-lowering hypothesis should be regarded as unresolved rather than refuted, and the design differences recorded here are what will make the next four readouts interpretable when they arrive.
